# International Contributions to Medical Literature. "Festschrift" in Honor of Abraham Jacobi, M.D., LL.D., to Commemorate the Seventieth Anniversary of His Birth, May 6th, 1900

**Published:** 1901-03

**Authors:** 


**International Contributions to Medical Literature. "Festschrift" in Honor of Abraham Jacobi, M.D., LL.D., to Commemorate the Seventieth Anniversary of his Birth, May 6th, 1900.** Pp. xiii., 496. New York: The Knickerbocker Press. 1900.

The title of this book is taken from the German, as there is no word in the English language which is the exact equivalent of "Festschrift." It is the custom in Germany to present to an eminent clinical teacher, or professor especially distinguished in some branch of medicine or the cognate sciences, on his attaining his seventieth year, or twenty-fifth or fiftieth year of professorship, a volume of essays written by his former pupils, colleagues, and friends, to celebrate the interesting occasion and perpetuate his memory. Following this custom, the volume before us has been compiled in a similar fashion to celebrate Dr. A. Jacobi's seventieth birthday, and to show appreciation of his many and great services to the profession of medicine in the United States. Dr. Jacobi's name is most widely known and justly distinguished for his work in connection with the study and clinical teaching of the diseases of children; he was "the first to inaugurate systematic and special clinics in diseases of children in New York," and the first President of the American Pediatric Society. Not only distinguished as a teacher and a writer, Dr. Jacobi has "always exerted his influence towards improving the tone and morale of the medical profession," and has been in addition "an active and public-spirited citizen." The honour done to him by the publication of this book has been well earned during a long and distinguished career of many-sided usefulness, in which he has devoted himself to the advancement of all that is best in medicine. In a "Festschrift" it is a point of honour that all the contributors should give of their best, and this volume is no exception to the rule; it contains about sixty articles in English, French, and German. They deal with very various subjects; a large number are, as would be expected from the nature of Dr. Jacobi's own special work, concerned with diseases of infants and children, or upon subjects bearing upon these diseases. Amongst the authors are Adami, Baginski, Max Einhorn, Escherich, Crozer Griffith, Henoch, Hun, Keen and Spiller, Koplik, Willy Meyer, Osler, v. Ranke, Allen Starr, Sir Hermann Weber, and W. H. Welch. We would say, in conclusion, that the general excellence of the contributions is such as to render the volume worthy of a careful study, and that there are many reports of rare and interesting diseases which give it quite a special value, and are not to be found elsewhere. For the rest, it is well and clearly printed, and furnished with excellent illustrations and plates.

